# A new understanding and evaluation of food sustainability in six different food systems in Kenya and Bolivia

**DOI:** 10.1038/s41598-020-76284-y

**Published:** 2020-11-05

**Authors:** Johanna Jacobi, Stellah Mukhovi, Aymara Llanque, Markus Giger, Adriana Bessa, Christophe Golay, Chinwe Ifejika Speranza, Veronica Mwangi, Horacio Augstburger, Elisabeth Buergi-Bonanomi, Tobias Haller, Boniface P. Kiteme, José M. F. Delgado Burgoa, Theresa Tribaldos, Stephan Rist

**Affiliations:** 1 Centre for Development and Environment, University of Bern, Bern, Switzerland; 2 Institute of Geography, University of Bern, Bern, Switzerland; 3 Department of Geography and Environmental Studies, University of Nairobi, Nairobi, Kenya; 4 Geneva Academy of International Humanitarian Law and Human Rights, Geneva, Switzerland; 5 Institute of Social Anthropology, University of Bern, Bern, Switzerland; 6 Centre for Training and Integrated Research in Arid and Semi-Arid Land Development, Nanyuki, Kenya; 7 Comunidad Pluricultural Andino-Amazónica Para La Sustentabilidad, Cochabamba, Bolivia; 8 UNESCO Chair On Natural and Cultural Heritage and Sustainable Mountain Development, University of Bern, Bern, Switzerland

**Keywords:** Environmental social sciences, Agroecology

## Abstract

Food systems must become more sustainable and equitable, a transformation which requires the transdisciplinary co-production of knowledge. We present a framework of food sustainability that was co-created by academic and non-academic actors and comprises five dimensions: food security, right to food, environmental performance, poverty and inequality, and social-ecological resilience. For each dimension, an interdisciplinary research team—together with actors from different food systems—defined key indicators and empirically applied them to six case studies in Kenya and Bolivia. Food sustainability scores were analysed for the food systems as a whole, for the five dimensions, and for food system activities. We then identified the indicators with the greatest influence on sustainability scores. While all food systems displayed strengths and weaknesses, local and agroecological food systems scored comparatively highly across all dimensions. Agro-industrial food systems scored lowest in environmental performance and food security, while their resilience scores were medium to high. The lowest-scoring dimensions were right to food, poverty and inequality, with particularly low scores obtained for the indicators women’s access to land and credit, agrobiodiversity, local food traditions, social protection, and remedies for violations of the right to food. This qualifies them as key levers for policy interventions towards food sustainability.

## Introduction

The United Nations Sustainable Development Goals require food systems to become more sustainable and equitable. Achieving this entails not only securing people’s food supply, but also ensuring that food production, distribution and consumption are ecologically, economically and socially responsible, now and in the long term^1–3^. But this achievement still appears far off when considering that the predominant global food systems, while arguably highly productive, have not eradicated hunger and malnutrition^4,5^ and are continuing to overstep the planetary boundaries^6,7^.

Making food systems more sustainable requires integrative approaches. To transform them—i.e. to move beyond the classical focus of maximizing global food productivity^8–10^—requires co-creation of knowledge and action by social and natural scientists as well as non-academic actors^11,12^. This means engaging in transdisciplinary research and optimizing the complex interactions between food system activities—from production through consumption—to improve their social-ecological outcomes while maintaining functioning in the face of stress and shocks.

Addressing these challenges formed the core of a research initiative^13^ that co-created and applied a concept of food sustainability. We understood “food systems” as networks of actors and activities involved in food production, processing and storage, retail and trade, and consumption^14,15^. These networks include direct or indirect interactions with the natural resource base, governance context, and flows of information and services^16^. Food systems include agricultural systems^15^, which we take as an entry point. This understanding underpinned our analysis and comparison of sustainability of six food systems^17^, three in Kenya and three in Bolivia (Table 1). These two countries are among the few that have the right to food enshrined in their constitution. Both countries also have a high prevalence of undernourishment in the total population^4^. The selected cases are a sample of the diversity of food systems that is often obscured by analyses of national-level data^1,2,18^.

**Table 1:** Food system case studies in Kenya and Bolivia.

Location	Food system type^17^	Abbreviation	Characteristics
Bolivia (Santa Cruz Department)	Agro-industrial food system	B1	Soybean-based food system producing, storing, processing and distributing food and feed products in national and international markets
	Domestic–indigenous food system	B2	Diversified food system of the Guaraní people including maize, cassava, peanuts, fruits, peppers, beans and others. Most food produced is processed, stored and consumed within local households; surplus is locally sold or bartered
	Agroecological food system	B3	The “Agroecological Platform” is a local network of producers, processors and consumers under a jointly managed label for ecologically and locally produced food in and around the city of Santa Cruz de la Sierra
Kenya (Laikipia, Nyeri and Meru Counties)	Agro-industrial food system	K1	Horticultural companies produce vegetables for export, sometimes in outgrower schemes. Fresh produce is air-transported in refrigerated value chains to Europe
	Regional food system	K2	Food grains, milk and meat are produced and partly processed in Meru and Laikipia Counties, and retailed and consumed all over the country, e.g. in Nairobi, Rift Valley, Central and South East Kenya
	Local food system	K3	Characterized by short value chains of maize, potatoes, fruits and vegetables produced, sold and consumed in and around the local trading centres and townships notably of Nanyuki, Naromoru and Timau

Our food sustainability framework is based on a review of approaches that link food systems and sustainability^19^, amended through a transdisciplinary validation with key actors of different food systems. The resulting concept contends that a sustainable food system must simultaneously contribute to five dimensions: (1) food security^20^, (2) implementation of the right to food^21^, (3) reduction of poverty and inequality^22^, (4) high environmental performance^23^ and (5) social-ecological resilience^24^.

In this study, we ask: (1) What food sustainability indicators can provide relevant information about the five sustainability dimensions, and how do the selected food systems score? (2) How do different food system activities, ranging from production to consumption, contribute to overall food sustainability? (3) Across all six food systems under study, which of the indicators are most influential in determining whether a food system is sustainable?

The transdisciplinary co-creation approach resulted in a series of indicators (Table 2), a broad base of empirical knowledge on food sustainability, rating criteria, and consensus on sustainability scores (Supplementary Data). The indicators were assessed by five interdisciplinary research teams, organized along the five dimensions of food sustainability. Aggregating the scientific evidence on the five sustainability dimensions for the six food systems in one database enabled us to define an overall score, providing a “big picture” of food sustainability. We then identified trends across the six food systems for a dialogue with food system actors, including policymakers, on key levers to simultaneously amend the systems’ negative features and strengthen the positive, in turn increasing overall levels of food sustainability.

**Table 2:** Indicators of food sustainability (for detailed indicator descriptions and results, see Supplementary Data). Citations indicate scientific publications applying these indicators.

Dimension	Description	Indicators	Food system activity
Food security^20,25^	Access, availability, utilization, and stability of food	Access to land by food system actors	Production
		Access to water for production	Production
		Capacity in the food system to process food	Processing and storage
		Capacity to store food	Retail and trade
		Availability of food at affordable prices	Consumption
		Share of locally produced food in the food system	Consumption
		Ability to provide food to food system actors	Consumption
		Capacity of the food system to fulfil the perceptions of local families of a “good diet”	Consumption
		Household food security level	Transversal
Right to food^21^	Implementation of the state’s obligations to respect, protect and fulfil everyone’s access at all times to adequate food or means for its procurement	Water accessibility for domestic consumption	Production
		Water quality for domestic consumption	Production
		Food system’s impact on overall water accessibility for irrigation	Production
		Access to seeds	Production
		Perceptions on land tenure/land rights	Production
		Proportion of women with land rights (access, use and tenure of land)	Production
		Proportion of women who have access to agricultural credit	Production
		Contribution to food diversity	Consumption
		Covering nutritional needs	Consumption
		Promotion of local food traditions	Consumption
		Perception on access to food-related information	Consumption
		Perception on participation in decision-making related to food	Consumption
		Remedies for violations of the right to food	Transversal
		Child labour (proportion of school age children not engaged in work in the food system)	Transversal
Poverty and inequality^24,26–28^	Distribution of incomes and assets along value chains	Farmers’ incomes	Production
		Wages of large-farm employees	Production
		Wages of employees at processing and storage levels	Processing and storage
		Wages of employees at retail level	Retail and trade
		Food expenditure and consumption baskets (including non-marketed production)	Consumption
		Financial capital (savings, income, access to finance); Human capital (education, experience, health)	Transversal
		Social capital: membership or participation in networks; mutual support; use of group tools/equipment/infrastructure	Transversal
		Physical capital (infrastructure, fulfilment of basic needs, material necessary for the system to function (e.g. transport, storage facilities) livestock	Transversal
		Natural capital: quantity and quality of households’ natural resources	Production
		Decent and safe working conditions	Transversal
		Social protection: access to social security, health care and income security	Transversal
Environmental performance^29–32^	Effects of food systems on the quality of the natural resource base and the wider environment	Agroecosystem service capacity	Production
		Soil quality	Production
		Use of agrochemicals	Production
		Use of materials (plastics and others)	Transversal
		Energy use intensity	Transversal
		Carbon footprint	Transversal
		Water footprint	Transversal
		Health impact perceptions related to the food system	Transversal
Social-ecological resilience^23,24,33–35^	Resilience of the food system in terms of buffer capacity, self-organization, and the capacity for learning and adaptation	Diversity of crops and breeds	Production
		Landscape heterogeneity	Production
		Liveable wage	Transversal
		Decentralization and independence	Transversal
		Local consumption of production (proportion of food that is produced and consumed locally or on-farm)	Consumption
		Organization in interest groups	Transversal
		Ecological self-regulation (provision of habitats for biodiversity)	Production
		Connectivity of food systems and their components	Transversal
		Knowledge of threats and opportunities	Transversal
		Reflective and shared learning	Transversal
		Functioning feedback mechanisms	Transversal
		Knowledge legacy and identity	Transversal
		Shared vision on the food system	Transversal

## Results

### Food sustainability indicators

The indicators of the five dimensions of food sustainability that were collectively defined and assessed in the six food systems are presented in Table 2 and in the Supplementary Data (sheets 1–9). Relevant across contexts, the indicators represent a consensual output of the research process with scientists from the Global North and South and non-academic actors related to the different food systems (see Methods). The indicators cover different activities, from production to consumption, and some are transversal, i.e. occurring along the value chain.

### How the six food systems scored

Food systems B3 (Agroecological food system) and K3 (Local food system) had the highest overall sustainability scores. In addition, these scores were more equally distributed across the five dimensions than in the other food systems. The greatest contributor to these high scores was environmental performance: both food systems demonstrated a high capacity to provide agroecosystem services (e.g. through crop diversity or combining livestock with trees^29,33^); low external inputs and recycling of organic materials; a low carbon footprint; and perceived positive health impacts by producers, workers and consumers. The food system that scored highest (4.0) in environmental performance is the Domestic–indigenous food system (B2). However, it obtained the lowest scores in poverty and inequality (1.6, with particularly low ratings for incomes, livelihood capitals and social protection), pulling down its overall score.

**Figure 1:**
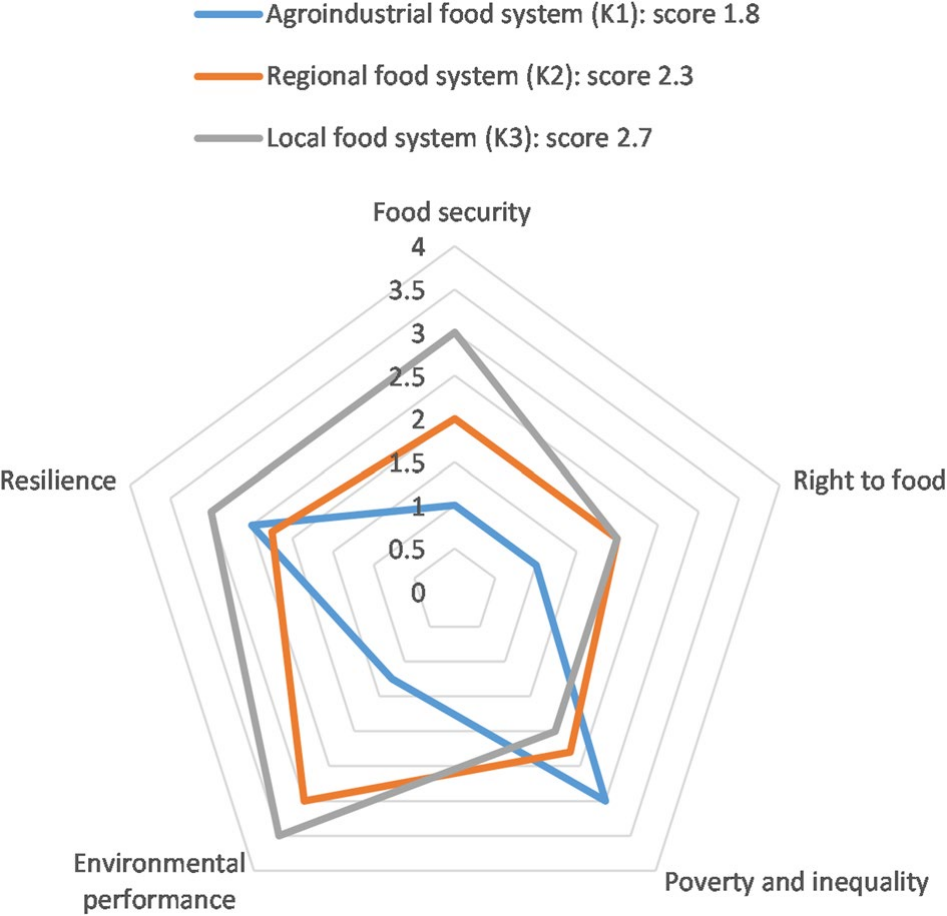
Overall food sustainability scores and median scores of five dimensions for three food systems in Kenya, rated from 0 (very low), 1 (low), 2 (medium), 3 (high) to 4 (very high). For detailed results, see Supplementary Data.

**Figure 2:**
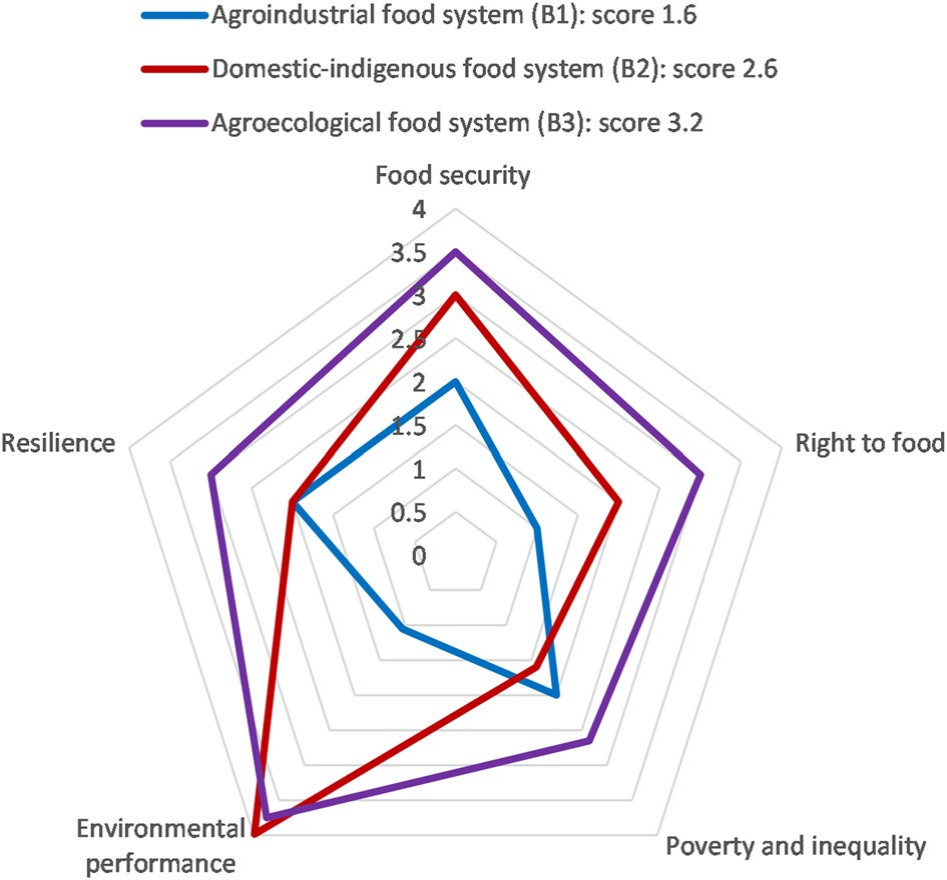
Overall food sustainability scores and median scores of five dimensions for three food systems in Bolivia, from 0 (very low), 1 (low), 2 (medium), 3 (high) to 4 (very high). For detailed results, see Supplementary Data.

Figures 1 and 2 display the aggregated qualitative and quantitative research results on a five-point Likert scale from 0 (very low) to 4 (very high). The area covered by one food system reflects its overall sustainability, while the axes reflect the five dimensions. The median is calculated as an average value for one dimension from all its indicators; for each food system it represents strengths (comparatively high scores) and weaknesses (comparatively low scores) of food sustainability of the six assessed food systems.

The lowest overall sustainability scores were obtained by the Agro-industrial food systems, B1 (scoring 1.6) and K1 (scoring 1.8). This was mainly due to their poor environmental performance on pesticide and resource use. Of the pesticides documented during this study, 65% in Bolivia and 67% in Kenya contained substances considered “highly hazardous” jointly by the FAO and WHO^36^. Additionally, resource use along the value chain was high, with examples including water, packaging material, electricity and diesel, and, in Kenya, aviation turbine oil^30,37^. Lowest-scoring B1 demonstrated a low diversity of crops and breeds, high greenhouse gas emissions and perceived negative health impacts. Right to food was particularly low in B1 due to low quality and accessibility of land and water resources for the local population, low food diversity and access to seeds, low access for women to land and finance, and a lack of participation in decision-making. In second-lowest scoring K1, water use was around 100 times higher than in K3, pesticide use seven times higher, and the carbon footprint of exported vegetables 67 times higher than for vegetables consumed in K3^30^.

Food security of local households in the study areas was highest in the Agroecological food system (B3), with better scores than the other food systems for access to land and water, contribution to local consumption, accessibility of food, and capacity to provide what is considered to constitute a “good diet”. In general, household food security was high in the study area in Bolivia, and low to medium in the study area in Kenya. Food security was lowest in the Agro-industrial system in Kenya (K1). This is because K1 exports almost all the food it produces and does not engage in processing or storage activities, implying low accessibility to, and consumption of, the produced food locally. Households involved in K1 through labour had medium food security and a low perception of the food system’s capacity to provide a “good diet”.

Contrary to expectations, the Agro-industrial food systems obtained medium (B1) and above-medium (K1) resilience scores. Key factors were a high or very high level of self-organization in interest groups, knowledge on threats and opportunities, and functioning feedback mechanisms between system components, such as supportive policies that translated into subsidies, relief payments and reduced tax rates^24^. This social dimension of resilience somewhat mitigated the low scores that B1 and K1 obtained for agroecosystem resilience and their high dependence on external inputs and monocultures (which, in turn, rendered them vulnerable to e.g. climate impacts or price fluctuations).

### The weakest dimensions across food systems

The weakest dimension was right to food. K1 and B1 both scored particularly low in this dimension due to high land concentration (e.g. average land plot size was 90 ha in K1, compared to 2 ha in K3^24^) and a lack of food diversity, supply of nutritional needs, and local food traditions. All food systems obtained low scores for women’s access to land and credit (in Kenya, only 5% and in Bolivia 17% of landowners are women^38^). K3 obtained slightly higher scores, as more women had access to land (although this did not mean they held the property deeds) and because of the prevalence of women’s groups that operated a system of microcredits.

The second-weakest dimension was poverty and inequality. This was due to low farming incomes and high income inequality (e.g. salaries for selling agricultural inputs in B1 were 220% higher than for the other activities in this food system^24^). Salaries for workers (e.g. farm workers in Kenya^39^) were around the minimum wage, and workers throughout the value chain were excluded from social protection. Nevertheless, the Agro-industrial food systems obtained a high (K1) and a medium score (B1) for the reduction of poverty and inequality, due to high scores for physical capital (infrastructure, fulfilment of basic needs, transport and storage facilities, livestock) and human capital (education, experience, health), and relatively low household expenditure on food.

### Contributions of food system activities to sustainability

**Figure 3:**
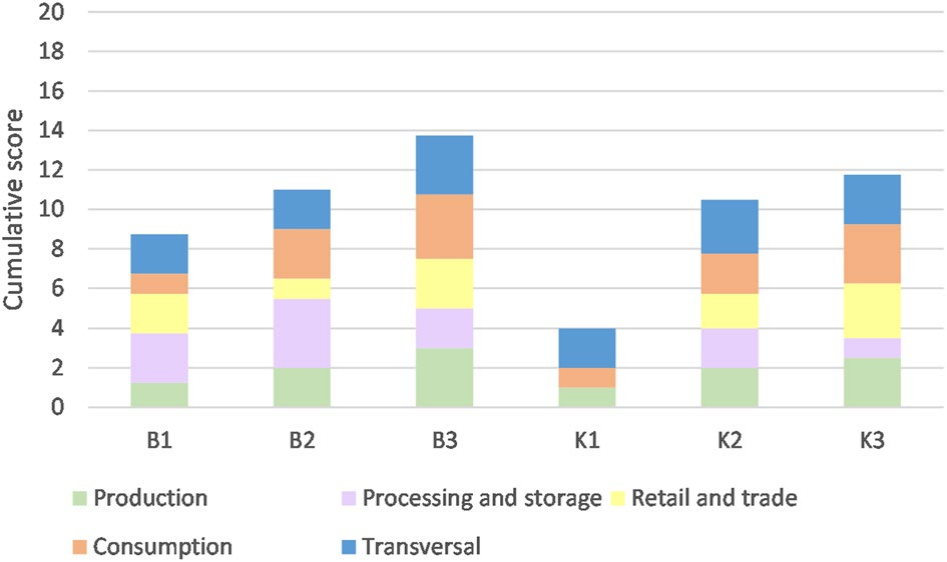
Median food system activity score of food sustainability. “Transversal” means across all food system activities. The maximum score for each food system activity is 4 (or “very high” on the Likert scale), and the overall maximum score is 20.

**Figure 4:**
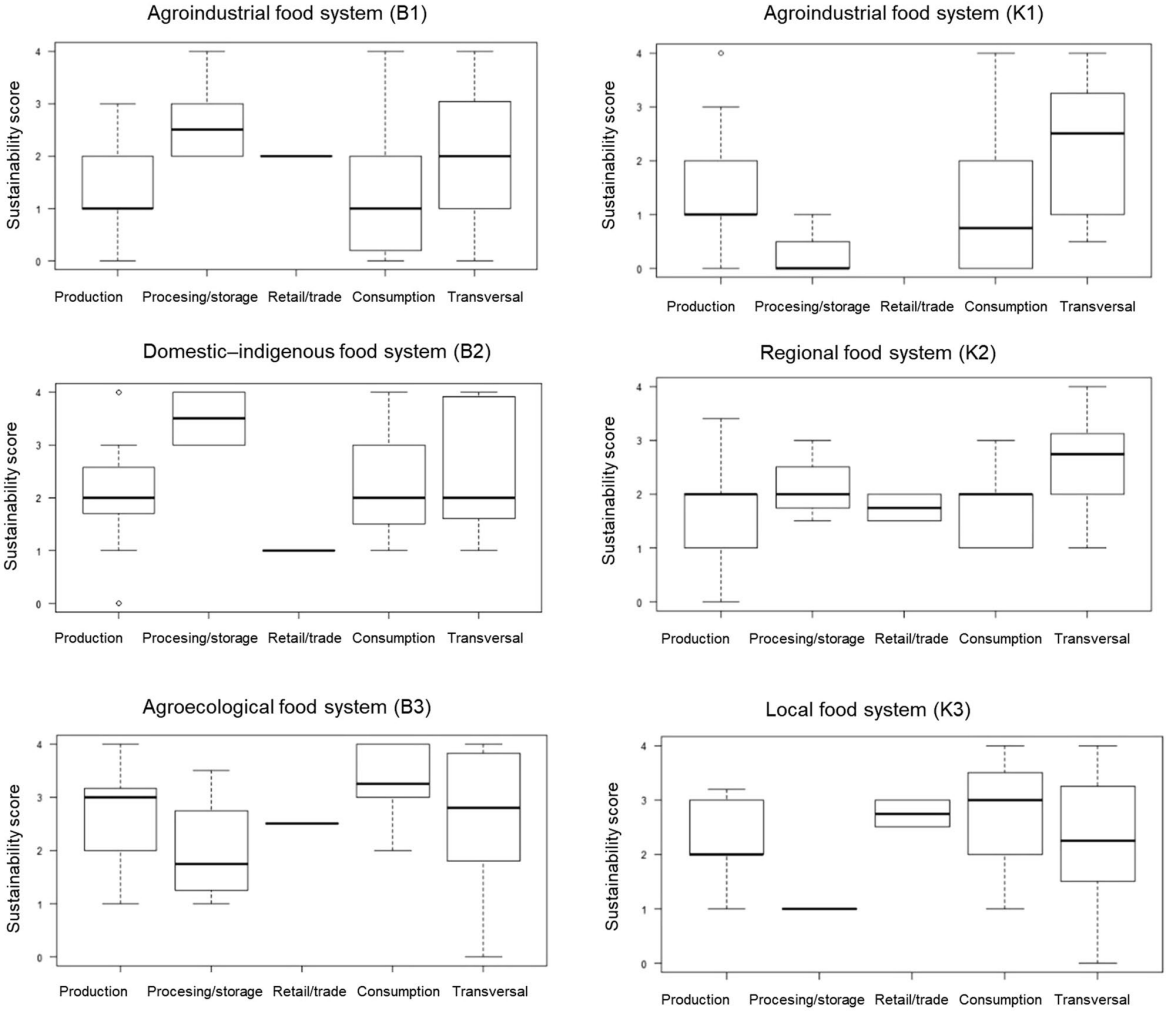
Distribution of sustainability scores for each food system according to food system activity: production, processing/storage, retail/trade, consumption and transversal indicators.

To understand the contribution of different food system activities to the overall sustainability scores, the indicators for each food system are grouped according to activity: production, processing and storage, retail and trade, consumption, and transversal (across activities, e.g. carbon footprint of a food product). Figure 3 shows the sum of the medians according to activity, and Fig. 4 shows the range of scores for each activity in each food system.

In the food systems with a comparably high overall sustainability score (B3, K3), all activities obtained relatively high scores (e.g. consumption in B3: locally produced food, provision of food to food system actors, a perceived “good diet”, contribution to food diversity, information and participation). The “transversal” category recorded similar scores across food systems. It comprised household food security, livelihood assets, material and energy use along the value chain, and resilience indicators (e.g. organization in interest groups, also along the value chain). The food system with the lowest cumulative score, K1, scored 0 in processing/storage and retail/trade, and it obtained low scores for production (due to low incomes), access to productive resources, environmental performance, and consumption (due to low contributions to the local food system and its diversity). Transversal scored higher than the other activities in K1, mainly due to the positive social resilience scores mentioned above. Figure 4 shows the per-activity contribution to the overall food sustainability rating for each food system.

Most food system activities (especially production, consumption and transversal) had a high variability of scores, ranging from 0–3 or even from 0–4 (minimum to maximum value). In B3, every activity obtained a comparably high score, although all but retail and trade were still very variable. Processing and storage (capacity in the food system to provide both processing and storage) was medium to high in B2, but storage was low in K3 and B3 (weakening overall food security) and K1 (freshly sold perishable produce). Retail and trade (affordable food prices, above-medium retail employee wages) contributed strongly to overall food sustainability in B3 and K3, at a medium level to B1 and K2, and little to B2. Consumption obtained a medium or above-medium score, which means that it played an important role in overall sustainability (e.g. in the form of food diversity in K3). An exception was K1, where consumption took place so far away that most of the related indicators obtained low scores for the food system context under study. Scores obtained for the “transversal” category also varied highly, but augmented overall food sustainability mainly through resilience (K1, B1) and environmental performance indicators (K2, K3, B3, K3).

### Most decisive indicators for food sustainability

**Figure 5:**
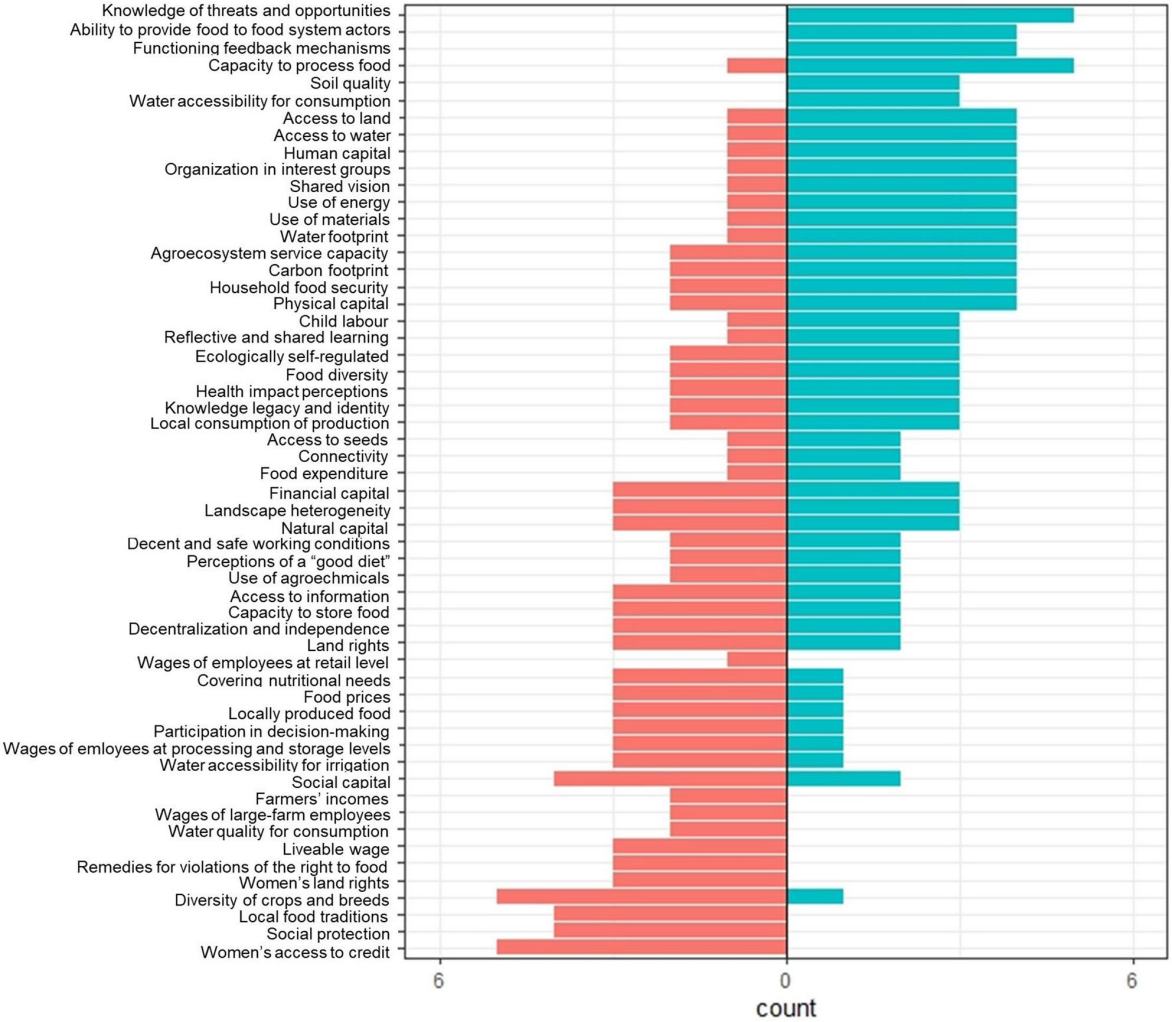
Frequency of difference from the median (to the left of 0: frequency with which the indicators across all six food systems scored worse than the median; to the right of 0: frequency with which they scored better than the median).

To identify general trends, we further analysed the importance of individual indicators for overall food sustainability across all six food systems (Fig. 5).

Resilience indicators often had a strongly positive influence on overall sustainability, especially the knowledge of threats and opportunities indicator, with above-median scores in five of the six food systems, and the indicators on functioning feedback mechanisms, interest groups and shared vision, which achieved above-median scores in four of the food systems. A notable exception was diversity of crops and breeds, a resilience indicator which scored lower than the overall median in five food systems. Several indicators from the food security dimension scored better than the median in four food systems: ability to provide food to food system actors, capacity to process food, access to land, access to water, and household food security.

In addition, environmental performance indicators were often high (e.g. use of energy, soil quality, use of materials, water footprint, Agroecosystem Service Capacity Index, carbon footprint), but mainly for the more local and diversified food systems. Exceptions were formed by the water footprint and use of materials along the value chain or food system stages, which were low also in B1 (as calculated up to the first consumption stage, e.g. use of soybeans for feed in meat and dairy production).

Low-scoring dimensions—those that pulled down the overall food sustainability score, i.e. poverty and inequality, and right to food—included indicators that most frequently scored lower than the median. These were related to gender, dwindling agrobiodiversity and food diversity, and precarious work conditions at the production level. Of these, women’s access to credit and diversity of crops and breeds scored five times below the median. The second-worst indicators (four times below the median) with no positive score were social protection and local food traditions, and the third-worst were the proportion of women with land rights, remedies for violations of the right to food, and liveable wage.

A principal component analysis (PCA) providing information on which combinations of indicators are most decisive for overall food sustainability in our case studies confirmed the trend shown in Fig. 5. Four principal components retained based on their eigenvalues explained 99% of variance (Supplementary Table S1). By retaining indicators with component loadings > 0.45, the first principal component was most influenced by human capital, social protection, remedies for violations of the right to food, local food traditions, access to information, landscape heterogeneity, water quality for domestic consumption, and women’s access to credit. Most of these indicators belong to the right to food and poverty inequality dimensions and are related to diversity and quality of human and natural resources that households, and especially women, have access to. The second principal component was most influenced by the capacity to process food, accessibility of water for domestic consumption, farmer incomes, ability to provide food to food system actors, use of materials, use of energy, and the capacity to cover nutritional needs, and was thus mainly linked to environmental performance and food security. The third principal component was dominated by resilience indicators: interest groups, knowledge of threats and opportunities, decent and safe working conditions, use of energy, shared vision, and ecological self-regulation. The fourth principal component was mostly influenced by access to water for domestic consumption and for irrigation, wages in retail, household food security, the proportion of women with land rights, and reflective and shared learning, and was thus strongly related to access to resources and incomes, particularly for women.

From the two analyses (frequency of positive/negative scoring of indicators, and PCA), we can identify the indicators with the greatest influence across the food systems under study. Six of these contributed positively, meaning that they were in a rather good state in several of the food systems. Four of the six indicators were from the food security dimension (capacity of the food system to process food, ability of the food system to provide food to food system actors, household food security, access to water) and two were from the environmental performance dimension (use of materials and use of energy). We identify six indicators, all but diversity of crops and breeds from the right to food dimension, which had a strongly negative influence (women’s access to credit, social protection, local food traditions, women’s land rights, and remedies for violations of the right to food). This means that these indicators were in an undesirable state in most of the food systems.

## Discussion

We assessed the sustainability of six food systems in Bolivia and Kenya using a novel, co-created framework combining five dimensions: food security, right to food, poverty and inequality, environmental performance and social-ecological resilience. We found that the food systems of highly diversified family and smallholder farms (B3, B2, K3) had the highest overall sustainability scores. In second place is the Regional food system in our study area in Kenya (K2), which is less diversified and operates larger, more specialized farms and value chains than the first group. Finally, the least sustainable food systems were Agro-industrial food systems (B1, followed closely by K1). These are operated on comparatively large scales and are heavily specialized and capitalized. The dimension that differed most among food systems was environmental performance. The dimension with the lowest scores was the right to food, which achieved a high value only in the Agroecological food system (B3).

Comparing food sustainability scores of different food system activities reveals that the more sustainable food systems obtained medium to high scores for processing and consumption. This underlines the importance of going beyond the classical focus on production^8^ and instead addressing food sustainability issues as part of a food system approach, from production to consumption and beyond^10,14^.

Recent food system research has developed and applied multi-dimensional assessments, some of which cover sustainability dimensions similar to those examined in our study^1,2,40^. However, the dimensions used generally apply to national levels and thus do not account for—often very pronounced—differences in production, processing, distribution and consumption at the subnational or local levels. It is notable that participatory and transdisciplinary food system assessments with a view to transformation to more sustainability and, particularly, to higher degrees of resilience, have also been emerging^41,42^. Acknowledging that both approaches—at national and at subnational levels—are important but need to be connected, our study bridges the gap between generating a broad data set covering a range of contexts while capturing the richness and depth of context-specific empirical data.

The principal component analysis and frequency of positive/negative ratings across food systems helped identify the indicators that were most decisive for overall sustainability. Most of the indicators refer to social aspects of food sustainability. This means that social policies can increase food sustainability, especially in combination with policies that improve agrobiodiversity and the environmental performance of the systems^43,44^. Policies based on a food system approach should therefore recognize that combining social and environmental policies hold higher potential than narrowly defined sectoral policies^45,46^. This becomes even more evident if we consider that some of the most critical indicators of food sustainability revolve around women’s access to land and credit, low agrobiodiversity and vanishing food traditions. These issues are exacerbated by low wage levels, exclusion from social protection, and a lack of remedies for rights violations.

These indicators are thus possible levers for food sustainability that need increased attention from policymakers. Focusing on women is imperative, not only to implement the right to food and reduce poverty and inequality^21,47^, but also because it may help increase biodiversity on farms and plates, and subsequently, resilience of the food system to stress factors^48,49^. Implementing the right to food means to respect, protect and fulfil people’s ability to produce or purchase food^21,50^. This refers, in particular, to strengthening women’s positions in food systems, and to focusing on the promotion of food diversity and agrobiodiversity^51^. The 2018 UN Declaration on the Rights of Peasants, approved by 119 countries, prominently includes these aspects^52^.

Finally, the transdisciplinary process of knowledge co-creation on food sustainability can enable food systems to learn from one another. For instance, job creation as often reported as a positive impact of agro-industrial food systems can also take place in more sustainable food systems^53,54^. An important conclusion from our assessment is that a high score in only one dimension or in only one food system activity cannot lead to overall food sustainability if other dimensions or activities are weak. For instance, while the resilience (or aspects of resilience) of a specific food system may be high, this alone may not be desirable if other sustainability dimensions are weak. Food systems with low scores in other dimensions such as environmental performance, must therefore be limited in their predominance and expansion, e.g. through better regulating the need to prevent social-ecological damage, and more sustainable food systems must be strengthened by redirecting land, resource, trade and investment policies from low-scoring to high-scoring food systems. Taking a multidimensional perspective of food sustainability, and identifying food system-specific strengths and weaknesses, is essential to inform policies that aim at making entire food systems—not only fragments of them—more sustainable^55^. This may help to avoid trade-offs between conflicting policy objectives, such as promoting productivity at the expense of social-ecological impacts.

## Methods

### Concept and case studies

Two workshops among the project partners (two universities in Switzerland, two universities and one non-academic partner organization each in Kenya and Bolivia) were held to define the concept of food sustainability, and to select study regions meeting the following criteria: (1) representing different types of food systems^17^; (2) representing case studies in an area crucial to the national food supply; (3) allowing the study of conflicts, competition and synergies in the context of interacting food systems. These criteria led us to select six food systems located in the Bolivian department of Santa Cruz and the region north-west of Mount Kenya: Agro-industrial (K1 and B1), Regional (K2), Local (K3), Domestic–indigenous (B2), and Differentiated-quality (here: Agroecological) (B3) food systems (Table 1).

### Indicators and aggregation of results

For each dimension of food sustainability, we identified and discussed indicators in three transdisciplinary workshops in Kenya and three in Bolivia with 11–25 participating scientists from the Global North and South, and 5–8 non-academic actors (civil society organizations, government representatives and private sector representatives) from the Global South. In Kenya, this included participants from the University of Nairobi, Aga Khan University, University of Bern, Geneva Academy for International Humanitarian Law and Human Rights, University of Bonn, Centre for Training and Integrated Research in Arid and Semi-Arid Land Development (CETRAD), Africa Agri-business Academy, Kenya Agricultural and Livestock Research Organization, and two participants from the Agricultural Sector Development and Support Programme (Isiolo and Meru Counties). In Bolivia, this included the Universidad Mayor de San Simón -Cochabamba, Universidad Autónoma Gabriel René Moreno—Santa Cruz, Probioma, the Ministry of Environment and Water, and the same European universities as in Kenya. While the number of indicators per dimension varied (Table 2), we applied the same indicators to all food system case studies, ensuring that there was no bias between the dimensions in the evaluation. Five interdisciplinary research teams investigated these indicators in Kenya and Bolivia from February 2015 to December 2017. In order to aggregate the abundant empirical qualitative and/or quantitative data obtained for each indicator, we organized an iterative, transdisciplinary evaluation process using a five-point Likert-scale. Each indicator was assessed on a scale ranging from 0–4 (“undesirable” to “desirable”, or 0–100%)^56,57^ in order to combine the qualitative and the quantitative data. The rules and criteria for the ratings were developed and the rating conducted during three-day workshops in Bolivia and Kenya by the local research teams and the international research team. The rating process included the same academic actors as the definition of the indicators, plus the non-academic project partners (CETRAD in Kenya and Probioma in Bolivia). Activities were: (1) revision of the indicator list according to empirical knowledge (retaining those that were assessed in all six cases for the present study); (2) contextual definition of rating criteria for each indicator on the Likert scale, to allow inclusion of quantitative and qualitative results; (3) rating of each indicator according to the empirical results on the six food systems, based on a critical interdisciplinary validation of the information about each indicator. We used official benchmarks where possible (e.g. minimum wage), and where this was not possible (e.g. diversity of crops and breeds), we used the maximum value found in each context as the 100% value. For qualitative indicators, we discussed with the study participants what situations were “desirable” and “undesirable”. For all rating criteria and ratings see Supplementary Data (sheets 1–9). Overall food sustainability was obtained for each food system by calculating an arithmetic mean of the median values for each dimension.

### Data sampling methods and analysis

We explain below the methods used for the empirical assessment of each dimension. Information was obtained from food system actors whose livelihood systems are directly related to the main value chains of the food system under analysis.

The transdisciplinary processes and our data aggregation approach contain three possible limitations. First, the integration of several sub-studies with locally adapted methods, and eventually the integration of quantitative and qualitative data on a common scale, implies challenges for further analysis (e.g. non-equal distances between the rating values preventing parametric testing and averaging). However, the added value of, for instance, deepening food security surveys with participant observation, makes data sets more valuable and meaningful than without such combinations^58^. A second challenge of comparing different food systems is that indicators are not of equal importance to food system actors in different contexts. For this study, we reduced the set of indicators from originally over 70 to 56 (Table 2), using only those indicators that were assessed in all six contexts, indicating their relevance in all these contexts. Third, the rating and aggregation of results by an expert panel can insert bias^59^. We argue that the empirical data combined with expert knowledge of a transdisciplinary team provides a chance for less bias than from a single researcher or disciplinary team, aware that aggregation of a complex data set implies the simplification of information. For this reason, we provide the original results for each indicator for each food system as well as the rating criteria and the aggregated results in the Supplementary Data sheets.

### Food security

We understand “food security” to be broader than availability, access, utilization and stability^60^ by integrating some of the key aspects relevant to self-sustained agricultural production, such as access to land and water.

#### Kenya

We conducted a survey on food security and livelihood indicators with 600 randomly selected households. A total of 65 households participated in the production process of large-scale agricultural operations that produce food for export in the international market (K1); 70 households participated in the production process of small, medium and large-scale agricultural farms that produce for the national food consumption and market system (K2); and 465 households participated in the production process of smallholder agriculture that produces for the local food consumption system (K3). Using the GoogleEarth base map of the open access GIS software QGIS, we first selected all houses (including farms) located at a distance of max. 15 km from an industrial farm to increase the probability of having a sufficient proportion of households participating in K1 production. The final randomized sample was selected using the random selection function of QGIS. Each selected house was given an ID. Ten enumerators were organized in groups of two people, to systematically survey the selected households. If no respondent was available at the time of the visit, the team revisited. If no one was living in the selected house, the enumerators surveyed the closest house to replace it. We also conducted 63 semi-structured interviews on household food security and livelihoods (21 in K1, 25 in K2 and 25 in K3, with different stakeholders from farmers to processors and traders)^27^. This included a 24-h memory of what the family consumed^61^. In K2, a short survey was conducted (N = 296: 8 input suppliers, 192 producers, 13 processors, 29 distributors, 33 retailers, 21 key informants)^22^. The survey covered costs and benefits, as well as food security^62^ along wheat, beef and dairy value chains. The data on the share of locally produced food in the food system came from the 77 resilience interviews (see social-ecological resilience below)^24^. A total of 50 semi-structured interviews and three focus group discussions with pastoralists^63^, 38 interviews and two focus group discussions with actors related to the horticulture industry (K1)^26^, and 32 semi-structured interviews with K3-related households provided additional, more in-depth information on food security indicators such as access to land and water, food availability, the capacity within the food system to process and store food, and perceptions on “good diets”^64^.

#### Bolivia

We conducted a survey on food security and livelihood indicators with 186 purposefully selected individuals (86 from B1, 50 from B2, 50 from B3); ten were input providers; 80 were producers; ten were involved in transport; three were in purchasing, 13 were in processing; ten were in retailing; and 60 were mainly involved in consumption^25^. All data were collected in the municipalities of San Pedro, La Guardia, Cabezas and Samaipata in the Santa Cruz Department. Data on household food production and its share in overall consumption came from 25 semi-structured and 16 open-ended interviews on agrobiodiversity, which included a 24-h memory of what the family consumed^61^. The data were complemented by an in-depth livelihoods study based on 23 semi-structured interviews on livelihoods assets (13 in B3, ten in B1) and by an ethnography based on participant observation in two Guarani communities^65^. To measure food insecurity, in Bolivia we used the “Escala Latinoamerciana y Caribeña de Seguridad Alimentaria (ELCSA)^66^, and for Kenya we used the “Household Food Insecurity Access Scale” (HFIAS)^67^, which is comparable to ELCSA. In both cases we used the lightest category (“in the past four weeks, did you worry that your household would not have enough food”) as an indicator for food insecurity.

### Right to food

Right to food indicators were assessed in workshops with research team members both from the Global North and the Global South, in Bolivia and Kenya (one in each country). In Bolivia, it included a human geographer, a rural sociologist, an environmental psychologist, and a right-to-food specialist. In Kenya, it included a social anthropologist, a legal expert, an economist, and a right-to-food specialist. Each indicator was analysed individually by the whole team, which—under the moderation of the right-to-food specialist—arrived at a consensus. The Kenyan assessment of right-to-food indicators was subject to further review by two specialists (a specialist in socio-ecological resilience and a specialist on the right to food).

### Poverty and inequality

#### Kenya

The survey on food security with 600 randomly selected households also covered livelihood indicators (see food security dimension, as the data were gathered together in the same survey). A survey on incomes, salaries and monthly expenditures in K2 (N = 296) took place within a value chain analysis^68^: eight input suppliers; 192 producers (small-scale farmers, pastoralists, large-scale farmers); 13 processors (millers, slaughterhouse workers, milk processors); 29 distributors (middlemen, traders, brokers); 33 retailers; 21 key informants along the value chains (wheat, beef and dairy value chains in K2 and K3)^22^. Similarly to the food security dimension, the poverty and inequality study was complemented with 63 household interviews on livelihoods^27^, and by the 38 semi-structured interviews and two focus group discussions with horticultural workers, representatives of trade unions, and managers in K1^26^. We also conducted direct observations in various channels (supermarkets, small stores, rural and urban formal and informal markets) for the overall assessment of the poverty and inequality dimension^69^.

#### Bolivia

Together with the food security assessment, our survey of 186 purposefully selected individuals from the three food systems in the Santa Cruz Department also provided data on poverty and inequality and, in this dimension, specifically on livelihood assets. The quantitative livelihood data were complemented by 23 semi-structured interviews (13 in B3, ten in B1) and participant observation in two communities in B2 (Pozo, in preparation)^28^. Perceptions on working conditions were gathered with seven open-ended interviews in B1 (farm workers, traders, drivers and input sellers)^23^.

### Environmental performance

#### Kenya

Data for life cycle inventories of key foodstuffs of the three food systems were collected through 111 semi-structured interviews, 11 narrative interviews and 29 questionnaires according to ISO 14,044 and covering the use of materials and energy, carbon footprint and water footprint^30^. A list of crop and breed diversity was obtained from 79 farms (three agro-industrial, four regional, 72 smallholder farms)^35^. Perceptions on health impacts related to work in and consumption of food from the different food systems was gathered by means of a questionnaire (380 households, five farm managers, 361 farm workers, 331 retailers), and structured interviews within this sample with 31 households, 30 farm workers, and 27 retailers^31^. More detailed information on perceptions on food and health came from 32 semi-structured interviews with local households^64^.

#### Bolivia

The research team conducted life cycle inventories according to ISO 14,044 of different key food products (Bascopé Zanabria, in preparation)^37^ (B1: soybean for national consumption, for export and for chicken production; B2: maize; B3; cabbage, onion, chard). This was done in 30 open interviews, 19 case studies of complete value chains including 19 surveys about energy use and material inputs; participant observation on 13 farms (nine in B1, one in B2 in a communal land, and three in B3); participatory mapping of landscapes and processes in nine cases (three in each food system)^23^. Perceptions on health impacts related to work in, and consumption of food from, the different food systems was gathered by means of 33 semi-structured interviews (nine government officials, eight NGO representatives and 16 farmers)^32^. The life cycle inventories were complemented from a more holistic perspective through an evaluation of the Agroecosystem Service Capacity Index (ASCI)^23,29^ in Bolivia and Kenya. The index calculates the capacity of farm-based agroecosystems to provide agroecosystems services. The index uses a list of 23 agroecosystem services ascribed to 99 possible land cover classes obtained from a participatory land cover mapping of the different agricultural landscapes related to a food system. The Agroecosystem Service Capacity (ASC) is calculated using the formula: ASC $= \left( {\frac{Si + Ni}{2}} \right)*Ai$ , where: Ai = percentage of the area occupied by the land cover class within an agroecosystem (e.g. a farm); Ni = the number of agroecosystem services the land cover can provide; and Si = the strength of a land cover class in providing each of the agroecosystem services^23^. The ASCI is then calculated using the formula: ASCI $= \mathop \sum \limits_{i}^{n} ASC$. This study uses an ASCI calculation from land cover mapping and agroecosystem services assessment on 9 farms (three K1, three K2 and three K3 farms). Soil quality (soil erosion status and % of bare ground) was evaluated using Visual Soil Assessment of FAO^70^.

### Social-ecological resilience

#### Kenya

Agrobiodiversity data came from lists elaborated on 79 farms (three in K1, four in K2, and 72 in K3)^35^. Participatory land use mapping was conducted on nine farms providing data on landscape heterogeneity^23^. The other resilience indicators were covered through 77 interviews in total: 25 smallholders; 20 pastoralists, five managers of horticultural companies; five retailers/middlemen, three wholesalers, and five restaurants; 14 organizations that deal with resilience building and risk mitigation (NGOs, a nutritional health expert, representatives of the national and county governments of Laikipia and Meru, relevant ministries, and research organizations)^24,33^. As in the case of the indicator for environmental performance, as the 100% value we took the highest value found in the sample each for Kenya and Bolivia. The data were complemented with information from the right-to-food workshops on liveable wages.

#### Bolivia

Diversity of crops and breeds was taken from 25 agrobiodiversity surveys and 16 semi-structured interviews^34^, providing both richness data and an average Shannon index for each food system. Further information came from the 23 semi-structured interviews (13 in B3, ten in B1) and participant observation in two communities in B2 mentioned under “Poverty and Inequality Bolivia” above. The indicator “liveable wage” was assessed from our food security and livelihoods survey of 186 purposefully selected individuals (see methods description for “Food security Bolivia” above). The data were complemented with information from the right to food workshops on liveable wages. The other indicators were assessed in 27 interviews with seven input suppliers, ten producers, three processing actors, four retailers, six consumers and eight actors who provided analysis and advice (NGOs and policymakers)^24^, and complemented by participant observation in seven cases (two in B1, two in B2 and three in B3)^71^, and participant observation on five agroecological farms in B3^72^.

Research data for all five dimensions were complemented through an ethnography within the food systems. This was carried out through participant observation of several months in and around one village in Kenya and one village in Bolivia^65,69^.

### Data analysis

Quantitative data came from local households and is thus applicable to the populations under study, but overall results are representative only of the specific food systems under investigation. Most households are small-scale food producers (and consumers), i.e. families whose livelihoods depend directly on the food system(s) to which they are connected. Measurements for the different sub-studies were taken from distinct samples. Interviews, field notes etc. were content analysed with Atlas.ti (Version 7.5.2), with both deductive and inductive codes. Survey data and final data integration were organized in Microsoft Excel (Supplementary Data). Statistical analyses were performed in R (i386 6.3.1). We used medians for the aggregation of the data because of their heterogeneity, and because the ordinal scale used does not allow the assumption of equal intervals between values. In Fig. 4 we display the results with interquartile ranges to indicate distribution. We performed a principal component analysis based on polychoric correlations on the Likert-scale data in psych package in R (fa.poly function) to identify the most influential indicators for food sustainability, based on their eigenvalues (Supplementary Table S1). We excluded three indicators that had N/A values (wages of large farm employees; wages of employees at processing, storage and transporting levels; wages of employees at retail level).

## Electronic supplementary material

Below is the link to the electronic supplementary material.

Supplementary material 1 (DOCX 18 kb)

Supplementary material 2 (XLSX 128 kb)

## Data Availability

The datasets generated and/or analysed during this study are available in the Zenodo repository, [https://zenodo.org/record/4009899#.X1RM2EF7lhE].
