# Supplementary material for: A new understanding and evaluation of food sustainability in six different food systems in Kenya and Bolivia
Source: Sci Rep. 2020 Nov 5;10:19145. doi: 10.1038/s41598-020-76284-y (PMC7645737; doi:10.1038/s41598-020-76284-y)
Supplement: Supplementary file 1 — Supplementary material 1 (DOCX 18 kb) [file 41598_2020_76284_MOESM1_ESM.docx]

**A new understanding and evaluation of food sustainability in six different food systems in Kenya and Bolivia**

Johanna Jacobi*^1,2^, Stellah Mukhovi^3^, Aymara Llanque^1^, Markus Giger^1^, Adriana Bessa^4^, Christophe Golay^4^, Chinwe Ifejika Speranza^2^, Veronica Mwangi^3^, Horacio Augstburger^1^, Elisabeth Buergi-Bonanomi^1^, Tobias Haller^5^, Boniface P. Kiteme^6^, José M.F. Delgado Burgoa^7^, Theresa Tribaldos^1^, Stephan Rist^1,2,8^

# ^1^Centre for Development and Environment, University of Bern, Switzerland. ^2^Institute of Geography, University of Bern, Switzerland. ^3^Department of Geography and Environmental Studies, University of Nairobi, Kenya. ^4^Geneva Academy of International Humanitarian Law and Human Rights, Geneva, Switzerland. ^5^Institute of Social Anthropology, University of Bern, Switzerland. ^6^Centre for Training and Integrated Research in Arid and Semi-arid Land Development, Nanyuki, Kenya. ^7^Comunidad Pluricultural Andino-Amazónica para la Sustentabilidad, Cochabamba, Bolivia. ^8^UNESCO Chair on Natural and Cultural Heritage and Sustainable Mountain Development, University of Bern, Switzerland.

*Correspondence: Johanna.jacobi@cde.unibe.ch

**Supplementary Table S1**. Principal component analysis of 56 food sustainability indicators

Principal Components Analysis

Call: principal(r = correlation$rho, nfactors = 4)

Standardized loadings (pattern matrix) based upon correlation matrix

RC1 RC3 RC2 RC4

Household_food_security -0.22 0.12 0.21 0.50

Access_to_land 0.13 0.00 -0.15 0.38

Access_to_water 0.14 0.13 -0.01 0.73

Capacity_to_process_food 0.16 -0.13 0.70 -0.05

Capacity_to_store_food -0.29 0.06 0.31 -0.02

Food_prices 0.30 -0.28 0.33 0.08

Locally_produced_food 0.10 0.03 0.02 -0.21

Ability_to_provide_food 0.06 0.41 0.57 0.08

Perceptions_on_good_diets 0.05 0.02 0.03 0.30

Water_accessibility_for_consumption 0.20 -0.34 0.62 0.13

Water_quality_for_consumption 0.48 -0.11 0.15 -0.22

Water_accessibility_irrigation 0.18 -0.05 -0.18 -0.67

Access_to_seeds 0.25 0.24 0.12 0.09

Land_rights 0.17 0.41 0.04 0.00

Women_With_land_rights 0.13 0.26 0.37 0.48

Women_access_to_credit -0.48 0.01 -0.11 -0.04

Food_diversity 0.10 0.43 0.14 0.16

Covering_nutritional_needs 0.24 -0.23 0.45 0.08

Local_food_traditions 0.57 0.01 0.35 -0.01

Access_to_information 0.55 0.36 0.05 0.24

Participation_in_decision_making -0.05 0.19 -0.01 0.12

Remedies_for_rights_violations -0.59 -0.26 -0.24 0.04

Child_labor -0.26 -0.06 -0.13 0.32

Farmers_incomes -0.14 0.13 0.59 -0.02

Wages_large_farm_employees -0.19 -0.04 -0.12 0.09

Wages_Processing_storage_transport -0.18 -0.06 -0.06 -0.04

Wages_retail 0.20 0.04 0.15 0.54

Household_food_expenditures 0.10 0.30 -0.07 0.34

Financial_capital 0.02 -0.07 -0.19 -0.10

Human_capital -0.64 0.14 -0.13 0.02

Social_capital -0.23 -0.41 -0.01 -0.02

Physical_capital 0.20 0.03 -0.32 0.20

Natural_capital -0.07 -0.44 0.09 -0.08

Decent_safe_working_conditions 0.26 0.46 -0.05 0.41

Social_protection -0.62 -0.22 0.15 0.03

Agroecosystem_service_capacity 0.37 0.29 -0.09 0.30

Soil_quality -0.38 0.19 -0.09 -0.25

Use_of_agrochemicals 0.29 0.14 0.33 -0.09

Use_of_materials 0.17 0.00 0.54 0.05

Use_of_energy 0.16 0.46 0.53 -0.27

Carbon_footprint 0.37 0.04 0.01 -0.22

Water_footprint 0.16 0.03 0.34 -0.07

Health_impact_perceptions 0.12 0.21 0.20 0.11

Diversity_crops_and_breeds -0.15 0.30 -0.14 0.11

Landscape_heterogeneity 0.50 0.20 -0.17 -0.04

Livable_wage -0.07 -0.26 0.34 0.07

Decentrailization_and independence 0.34 -0.11 -0.11 0.15

Local_consumption_of_production 0.43 0.28 0.07 -0.18

Interest_groups 0.25 -0.55 -0.22 0.26

Ecologically_self_regulated 0.18 0.45 -0.19 0.10

Connectivity -0.30 -0.01 0.04 -0.17

Knowledge_of_threats_and_opportunities 0.07 -0.49 -0.31 0.12

Reflective_shared_learning -0.18 -0.30 -0.19 0.48

Functioning_feedback_mechanisms 0.31 -0.43 -0.25 0.40

Knowledge_legacy_and_identity -0.05 0.52 0.09 -0.05

Shared_vision -0.08 -0.46 0.15 0.31

SS loadings 4.65 4.15 4.03 3.69

Proportion Var 0.08 0.07 0.07 0.07

Cumulative Var 0.08 0.16 0.23 0.30

Proportion Explained 0.28 0.25 0.24 0.22

Cumulative Proportion 0.28 0.53 0.78 1.00
